# Unnecessary ERCPs: Is Spontaneous Stone Passage the Sole Determinant?

**DOI:** 10.3390/medicina62030548

**Published:** 2026-03-16

**Authors:** Dimitrios I. Ziogas, Theodoros A. Voulgaris, Ance Volkanovska, Aliki Stamou, Georgios Kranidiotis, Gerasimos Stefanidis, Paraskevas Gkolfakis, Ioannis A. Vezakis, Gjorgi Deriban, Meri Trajkovska, Konstantinos Triantafyllou, Antonios Vezakis, Ioannis S. Papanikolaou

**Affiliations:** 1 Department of Gastroenterology, Athens Naval Hospital, 11521 Athens, Greece; dimiziog95@gmail.com (D.I.Z.); gkranidiotis@gmail.com (G.K.); gerstefanidis@gmail.com (G.S.); avezakis@hotmail.com (A.V.); 2 Department of Endoscopy, Second Academic Surgical Unit, National and Kapodistrian University of Athens, Aretaieion Hospital, 11528 Athens, Greece; thvoulgaris87@gmail.com (T.A.V.); aliceastam@gmail.com (A.S.); 3 University Clinic of Gastroenterohepatology, Faculty of Medicine, Ss. Cyril and Methodius University, 12345 Skopje, North Macedonia; ancevolkanovska@gmail.com (A.V.); gderiban@gmail.com (G.D.); meritrajkovska@gmail.com (M.T.); 4 Second Academic Department of Gastroenterology, Medical School, National and Kapodistrian University of Athens, 12462 Athens, Greece; pgolfakis@gmail.com (P.G.); ktriant@med.uoa.gr (K.T.); 5 Biomedical Engineering Laboratory, School of Electrical and Computer Engineering, National Technical University of Athens, Zografou Polytechnic Campus, 15772 Athens, Greece; ivezakis@biomed.ntua.gr

**Keywords:** endoscopic retrograde cholangiopancreatography, choledocholithiasis, spontaneous stone passage, endoscopic ultrasound, prediction algorithms

## Abstract

Endoscopic retrograde cholangiopancreatography (ERCP) is the cornerstone in the management of choledocholithiasis. Despite continuous advancements in technique and safety, ERCP carries a risk of significant complications, underscoring the importance of avoiding unnecessary procedures. The principal contributor to potentially avoidable ERCPs in patients with known choledocholithiasis is the spontaneous passage of common bile duct stones. Small stone size and a long interval between diagnosis and the procedure have increasingly been found to favor this event. Moreover, despite the development of well-defined risk stratification scores for patients with suspected choledocholithiasis, the incidence of negative ERCPs within this patient population remains considerable, even when a high suspicion of choledocholithiasis is evident. This review summarizes current evidence on the incidence and predictors of avoidable ERCPs in these contexts, with particular emphasis on spontaneous stone passage. It also discusses the role of endoscopic ultrasound (EUS) as a diagnostic tool to reduce unnecessary procedures when initial imaging fails to confirm the presence of stones despite persistent high clinical suspicion. By integrating and critically appraising recent findings, we provide practical guidance for clinicians on decision-making regarding ERCP, particularly in situations where spontaneous stone passage is likely or imaging results are inconclusive.

## 1. Introduction

Endoscopic retrograde cholangiopancreatography (ERCP) is an essential therapeutic tool for endoscopists in treating and preventing complications from common bile duct (CBD) stones. According to guidelines, performing ERCP is mandated in cases of cholangitis, biliary colic, and biliary pancreatitis, as well as in asymptomatic patients with CBD stones [1,2]. Unfortunately, ERCP incurs costs, exposes patients to the risk of multiple complications, and can even be life-threatening [3,4]. Therefore, it is of the utmost importance to have solid and unequivocal documentation confirming the presence of bile duct stones before proceeding.

Initial imaging modalities for evaluating suspected choledocholithiasis include abdominal ultrasound (US) and computed tomography (CT), while the most reliable techniques for this purpose are magnetic resonance cholangiopancreatography (MRCP) and endoscopic ultrasound (EUS). The latter demonstrates a slightly higher diagnostic ability compared to MRCP, although recent meta-analyses found no significant differences between the two modalities [5,6,7,8]. Unfortunately, in everyday clinical practice, it is not uncommon that when imaging suggests the presence of a stone in the biliary tree, subsequent ERCP fails to confirm it [9,10,11]. Such cases are frequently described in the literature as unnecessary ERCPs, although this terminology is not uniformly defined. This discrepancy may be attributed to spontaneous stone passage, false-positive imaging results, or the inability of the ERCP operator to visualize or document the stone during the procedure. Regrettably, for the latter two scenarios, there is no gold standard method to quantify their occurrence in clinical practice. Consequently, all such cases are often categorized under spontaneous stone passage. Several studies have examined the incidence of this event and its associated predictors, underlining its clinical significance. The likelihood of spontaneous stone passage has been shown to correlate with stone size [12,13]. Recent data also suggest that the timing of ERCP after a positive EUS for bile duct stones influences the outcome [14].

This review aims to shed light on the clinically significant issue of unnecessary ERCPs resulting from spontaneous stone passage by providing a detailed assessment of their incidence and contributing factors. Moreover, it does examine the occurrence of unnecessary procedures in another clinical scenario, specifically in patients with a high probability of choledocholithiasis but without stones detected on initial imaging. In addition to describing the clinical scenarios contributing to unnecessary ERCPs, this review also presents an evidence-based analysis of strategies to reduce their incidence. Together, these insights provide a holistic evaluation that could support clinicians in everyday practice.

## 2. Methods

This article was conducted as a narrative review of the literature. A search of the PubMed and EMBASE database was conducted for English-language studies published up to December 2025, using the following keywords: endoscopic retrograde cholangiopancreatography, unnecessary ERCPs, spontaneous stone passage, choledocholithiasis, endoscopic ultrasound, and prediction algorithms. A stepwise search strategy was applied, whereby keywords were searched in various combinations to identify studies addressing unnecessary ERCP. Priority was placed on meta-analyses, recent clinical trials, and prospective studies to ensure the inclusion of high-quality evidence. Study selection was based on relevance to the topic and the authors’ clinical judgment.

### 2.1. Incidence of Spontaneous Stone Passage

The incidence of spontaneous stone passage before ERCP has been investigated in multiple studies, highlighting the clinical significance of this event. In these studies, the reported range of spontaneous stone passage rates is 6% to 73% [12,13,15,16,17,18]. This wide variability can be explained by differences in study populations, imaging methods used before ERCP, and the indications and risk profiles of the patients included. For example, early studies involved patients in whom an intraoperative cholangiogram was performed during cholecystectomy for gallstones [15,17]. These studies reported an incidence ranging from 20% to 73%; however, they are limited by the lack of imaging confirmation of CBD stones before surgery. When using highly sensitive diagnostic modalities such as EUS or MRCP for detecting choledocholithiasis, the observed rate of spontaneous passage tends to be lower. For example, in reports like that of Frossard et al. [16], among patients with confirmed CBD stones who underwent subsequent ERCP after a positive EUS, the spontaneous passage rate was 21%. In a large recent study by Andreozzi et al. [12], which included patients with CBD stones diagnosed via multiple modalities (EUS, MRCP, CT), the spontaneous passage rate was 17.6%. Sperna Weiland et al. [19] reported a rate of 22% among 707 patients, while Saito et al. [13] observed a rate as low as 6.2% in their recent investigation. A comprehensive meta-analysis involving nine studies with a total of 3338 patients found a spontaneous passage rate of 15.7% [14].

### 2.2. Risk Factors for Spontaneous Stone Passage

Numerous studies have attempted to identify factors associated with spontaneous CBD stone passage (Table 1). In addition to stone size and number, patient age, CBD diameter, and the interval between imaging and ERCP have all been found to influence the likelihood of spontaneous passage.

### 2.3. Stone Characteristics

Stone size appears to be the most consistently reliable predictor of negative ERCP findings, as multiple studies demonstrate that smaller stones are more likely to pass spontaneously into the duodenum. This association is physiologically reasonable, as small stones can more easily traverse the sphincter of Oddi along with bile flow during coordinated biliary contractions. However, different cut-off points have been proposed. Frossard et al. [16] found that stones ≥8 mm had a 95% negative predictive value for spontaneous passage. A retrospective study of 96 patients reported that, in multivariate analysis, a stone size of less than 4.85 mm was the strongest predictor of spontaneous passage, with a sensitivity of 81.8% and specificity of 78.9%, respectively [20]. Saito et al. [13] in their large multicenter retrospective study found that stones measuring < 6 mm were significantly correlated with unnecessary ERCPs. Two other retrospective studies reported lower cut-off values of 4.3 mm and 4 mm, respectively, as predictors of spontaneous stone passage. (*p* < 0.001) [21,22]. Khoury et al. [23] reported an even lower stone size of 3.5 mm as a predictor, with 71% sensitivity and 69% specificity. A possible explanation for the slight variation in the reported cut-offs among studies could be the different imaging methods used to assess stone size across them. A meta-analysis confirmed that stones associated with spontaneous passage tend to be smaller, with a mean size of 3.7 mm, although no specific cutoff was established [14].

Regarding the number of stones, multiple studies show that a solitary stone has a higher tendency to pass spontaneously compared to multiple stones, though some findings diminish in multivariate analyses [13,20,21]. According to the Arabpour meta-analysis, having a single CBD stone is an independent predictor of spontaneous passage [14]. Moreover, a distal location, especially in the absence of significant CBD dilation, seems to facilitate the excretion of stones through the papilla [13,23].

### 2.4. Clinical and Laboratory Parameters

An important issue is whether clinical presentation can predict the likelihood of unnecessary ERCPs owing to the spontaneous passage of CBD stones. However, only a few studies have addressed this issue. Correia et al. [24] reported a retrospective study of 334 patients comparing those with spontaneous stone passage (*n* = 78) and those without (*n* = 256), and found that the former group was more frequently asymptomatic (59.0% vs. 33.6%, *p* < 0.001). In contrast, the presence of acute cholangitis significantly reduced the likelihood of stone migration (*p* < 0.05), possibly due to impaired biliary flow secondary to inflammation [24]. Moreover, few studies have shown that acute biliary pancreatitis is associated with unnecessary ERCPs due to stones moving spontaneously into the duodenum (*p* = 0.024) [12,24]. Indeed, spontaneous clearance has been observed in up to 80% of patients presenting with acute pancreatitis and evidence of CBD stones [25]. This might be related to the fact that acute pancreatitis is usually caused by small, distally located stones, which, as described earlier, are more likely to pass spontaneously through the ampulla. However, a recent meta-analysis found no significant relationship between clinical presentation and the occurrence of spontaneous stone passage [14]. Beyond clinical presentation, a correlation may exist between the clinical course of symptomatic patients and the chance of spontaneous passage of stones. In a retrospective study of 36 patients, Sakai et al. [26] found that ERCP was negative in all patients who showed clinical and laboratory improvement. Similarly, another retrospective study found that symptom relief predicted the absence of stones during ERCP in patients with acute cholangitis (*p* = 0.004) [20].

Regarding the role of laboratory parameters, the available evidence remains sparse. A retrospective study including 272 patients reported that improvement in gamma-glutamyl transferase (GGT) was a significant predictor of stone clearance, with a cut-off value of 408 IU/L yielding a negative predictive value of 89% for the absence of stones at ERCP [23]. Furthermore, Ding et al. [27], in a retrospective study, reported that GGT was inversely associated with stone migration in univariate analysis. In another retrospective study, patients with spontaneous stone passage had significantly lower bilirubin levels at imaging and before ERCP, compared to those without. Specifically, a pre-ERCP bilirubin level of ≤2 mg/dL was identified as the strongest predictor of unnecessary ERCP (OR = 9.224) [24]. The authors suggested that a decreased bilirubin level may reflect obstruction resolution, resulting in adequate biliary flow.

**Table 1:** Summary of Studies on Spontaneous Stone Passage.

Author and Year	Study Design	Number of Patients	Imaging at Diagnosis	Incidence (%)	Stone Size Threshold (mm)	Other Findings
Frossard et al. 2000 [16]	Prospective	92	EUS	21	≥8 mm predicted no spontaneous passage (negative predictive value 95%)	Acute pancreatitis associated with stone migration
Reid et al. 2017 [22]	Retrospective	221	MRCP	NA	>4 mm predicted positive ERCP, with a sensitivity of 83% and specificity of 66%	NA
Khoury et al. 2019 [23]	Retrospective	272	MRCP	NA	>3.5 mm associated with low chance of spontaneous passage (sensitivity 71%, specificity 69%)	Lower GGT (*p* = 0.023), less intrahepatic bile duct dilatation (*p* = 0.047), and distal stone location (*p* = 0.05) were associated with spontaneous passage; GGT ≥ 408 IU/L predicted no spontaneous passage (NPV 89%)
Sangualosit et al. 2020 [20]	Retrospective	96	US, CT, MRCP	19.8	>4.85 mm correlated with diminished chance of spontaneous passage (AUC 0.832, sensitivity 81.8%, specificity 78.9%)	Single CBD stone (OR 18.296) and symptomatic improvement (OR 11.091) were significant predictors of spontaneous stone passage
Ding et al. 2021 [27]	Retrospective	101	CT, MRCP	NA	<3.3 mm best predicted spontaneous passage	Lower total bilirubin, GGT, alkaline phosphatase, CA19-9, and α-L-fucosidase levels were associated with spontaneous stone passage (*p* < 0.05).
Andreozzi et al. 2022 [12]	Retrospective	1016	US, CT, MRCP, EUS	17.6	≤5 mm associated with unnecessary ERCP (OR 5.028, 95% CI 3.016–8.382, *p* = 0.000)	Pancreatitis (OR 2.775) was predictive of unnecessary ERCPS; female sex (OR 0.518) and diagnosis–procedure interval ≤7 days (OR 0.425) were linked with CBD stones at ERCP.
Saito et al. 2023 [13]	Retrospective	1260	US, CT, MRCP, EUS	6.2	<6 mm significantly increased the chance of spontaneous migration	Multivariate analysis: single CBD stone (OR 6.4), absence of CBD dilatation (OR 1.7), longer diagnosis–ERCP interval (OR 1.4) were associated with spontaneous passage.
Inan et al. 2024 [21]	Retrospective	236	MRCP	NA	≤4.3 mm predicted spontaneous passage (sensitivity 58%, specificity 85%)	Age (OR 0.973) and CCI (OR 0.747) were predictors of spontaneous stone passage in univariate analysis (none significant in multivariate analysis)
Correia et al. 2025 [24]	Retrospective	334	US, CT, MRCP	23.4	≤ 5 mm was significant predictor of spontaneous stone passage (OR 4.23, 95% CI 2.161–8.415, *p* < 0.001)	Multiple logistic regression: absence of acute cholangitis (0.237), bilirubin level ≤ 2 mg/dL (9.224) prior to ERCP predicted spontaneous stone passage

NA: not available, EUS: Endoscopic Ultrasound, MRCP: Magnetic Resonance Cholangiopancreatography, US: Abdominal Ultrasound, CT: Computed Tomography, ERCP: Endoscopic Retrograde Cholangiopancreatography, GGT: Gamma-glutamyl Transferase, CBD: Common Bile Duct, CCI: Charlson Comorbidity Index.

Although some retrospective studies suggest that asymptomatic presentation, improvement of symptoms, or normalization of laboratory parameters may be associated with spontaneous stone passage, the available evidence remains limited and heterogeneous. Importantly, the lack of association between clinical presentation and spontaneous stone migration in the meta-analysis by Arabpour et al. [14] underlines the inconsistency of current data. Therefore, clinical features should not be the sole factor in the decision to perform ERCP.

### 2.5. Timing to ERCP

Guidelines advocate for ERCP to be performed within 48–72 h after presentation in patients with acute cholangitis. However, no specific timeline has been established for asymptomatic individuals [2,28]. Moreover, in cases of mild cholangitis with prompt symptomatic improvement, or when acute cholangitis is accompanied by acute pancreatitis, the aforementioned timeframe is often extended [29,30]. That is, the timing of ERCP performance varies among studies, from as early as 6 h after confirmation of choledocholithiasis to as late as 30 days afterward. The interval between diagnosis and ERCP may influence the rate of unnecessary procedures, as a prolonged interval can increase the likelihood of spontaneous stone passage. This hypothesis was validated in a retrospective multicenter study, which found that patients without stones at ERCP had a significantly longer interval between imaging and the procedure, compared to those with stones (10.9 ± 22.0 vs. 4.8 ± 10.3 days; *p* < 0.001, respectively) [12]. It is noteworthy that this association was evident regardless of stone size. Moreover, the absence of stones was observed in 25% of patients who underwent the procedure within 7 days of diagnosis, increasing to up to 90% in asymptomatic patients who underwent ERCP after 90 days [12]. In another large retrospective study of 1260 patients, Saito et al. [13] reported that, irrespective of the presence of symptoms, the period from diagnosis to ERCP was significantly associated with spontaneous stone migration in multivariate analysis (OR 1.4, 95% CI 1.1–1.7; *p* = 0.012).

### 2.6. Other Factors

Conflicting results have been reported regarding the role of age [21,23]. While some studies suggest age does not significantly impact stone passage, the meta-analysis by Arabpour et al. [14] provided evidence that younger patients are more likely to experience spontaneous passage independently. A normal CBD diameter has been associated with a higher likelihood of spontaneous passage. Saito et al. [13] reported that nondilated CBD (<10 mm) is significantly linked to spontaneous stone passage. Khoury et al. [23] also observed this, indicating that the absence of intrahepatic dilation correlates with spontaneous passage. The Arabpour meta-analysis confirmed that larger CBD diameters inversely relate to spontaneous passage probabilities [14].

### 2.7. ERCP Complications

As outlined above, ERCP carries a significant risk of complications, namely acute pancreatitis, bleeding, infection, and intestinal perforation. Among these complications, acute pancreatitis is the most frequent, with a reported incidence ranging from 1.6% to 15% according to a recent review [3,31,32]. Bleeding, typically associated with sphincterotomy, occurs in 0.3–2% of procedures, while the risk of perforation is estimated at approximately 1% [31]. These adverse events may have a life-threatening course. Thus, minimizing unnecessary procedures is crucial. For instance, while guidelines recommend stone extraction for all CBD stone cases, this may not be the most appropriate or safe approach for asymptomatic patients with a small, solid CBD stone (0.3 cm) and a normal CBD caliber. Recent data suggest that ERCP in asymptomatic individuals may increase the risk of pancreatitis [33]. With respect to whether unnecessary ERCPs are associated with an increased risk of unfavorable outcomes, the existing evidence is sparse and contradictory. Correia et al. [24] reported that complications were more common in patients who underwent unnecessary procedures (8% vs. 4%), underlining the significance of predicting spontaneous stone migration. On the contrary, two recent multicenter retrospective studies from the same group found no difference in the rates of adverse events between patients with stones at ERCP and those without [13,34].

### 2.8. Unnecessary ERCPs in Suspected Choledocholithiasis

Another point that needs attention is the occurrence of unnecessary ERCPs in patients with suspected choledocholithiasis. To address this issue, both the European Society of Gastrointestinal Endoscopy (ESGE) and the American Society for Gastrointestinal Endoscopy (ASGE) published risk-stratification guidelines [2,28]. In cases of intermediate risk for choledocholithiasis, both guidelines advocate further imaging with EUS or MRCP for definitive diagnosis. ESGE considers high-risk patients as those with either a confirmed stone on initial imaging or features of acute cholangitis, whereas ASGE additionally includes a total bilirubin level exceeding 4 mg/dL combined with common bile duct dilation as a high-risk criterion. According to both guidelines, high-risk patients proceed directly to ERCP, even in the absence of bile duct stones on imaging [2,28]. Nevertheless, even among these patients, unnecessary ERCPs remain relatively common, with reported incidences ranging from 8% to 41% in the literature [35,36,37,38,39,40,41,42]. One should bear in mind that a portion of patients with negative ERCPs may indeed have had choledocholithiasis and experienced spontaneous stone passage. However, the reported incidence of negative ERCPs remains higher than that of spontaneous stone passage, as described earlier. Moreover, several studies have evaluated the diagnostic ability of the ESGE and ASGE high-risk criteria for choledocholithiasis and reported sensitivities of 64–79% and specificities of 56–99% for the ESGE criteria, and sensitivities of 71–84% with specificities of 53–97% for the ASGE criteria [11,39,40,43]. One possible explanation for this wide range is the differing nature of the high-risk criteria. Saito et al. [34] conducted a large multicenter retrospective study to assess the presence of bile duct stones during ERCP in patients with suspected choledocholithiasis who were classified as high risk according to ASGE criteria. Unsurprisingly, they found that patients with stones in the initial imaging had an excellent ERCP stone detection rate of 95.7%. In contrast, even in patients who met both clinical criteria, nearly half of the procedures were negative. Similarly, a recent meta-analysis consisting of 20 studies reported that the presence of stones on (or of a stone) US was the only reliable predictor of choledocholithiasis confirmed at ERCP [44]. Thus, performing ERCP solely based on clinical or laboratory parameters, without direct evidence of a stone, may result in a higher rate of unnecessary procedures. Beyond this aspect, Jagtap et al. [11] in their retrospective study, found that the ESGE criteria were more accurate than the ASGE criteria in predicting the presence of CBD stones on ERCP. They concluded that this difference might be attributed to the incorporation of bilirubin elevation combined with common bile duct dilation in the ASGE criteria, as, in their study, these features showed a lower positive predictive value compared to cholangitis [71.93% (95% CI 60.02–81.39) vs. 88.73% (95% CI 79.27–94.91), respectively]. In another retrospective study, total bilirubin >4 mg/dL plus CBD dilation demonstrated a similar PPV for choledocholithiasis of 78% [45]. Thus, the more restrictive nature of the ESGE criteria may lead to fewer unnecessary procedures by classifying more patients into the intermediate-risk group, where additional imaging is recommended [11,39]. In contrast, two recent studies found comparable accuracy between the ESGE and ASGE criteria in predicting choledocholithiasis [39,43]. Likewise, as seen with spontaneous stone passage, the presence of acute pancreatitis appears to reduce the likelihood of stone detection during ERCP in patients with suspected choledocholithiasis. Narváez-Rivera et al. [38] performed a prospective study and demonstrated that the absence of acute pancreatitis was significantly associated with stones in ERCP in patients with suspected choledocholithiasis (OR 3.23, 95% CI, 1.81–5.77). Notably, among 63 patients with ASGE high-risk criteria who also had acute pancreatitis, the rate of stone detection on ERCP was only 39.7%. Similarly, the negative predictive value of pancreatitis for the presence of stones on ERCP has also been confirmed in a recent meta-analysis [44].

### 2.9. EUS for Prevention of Unnecessary ERCPs in High-Risk Patients

**Figure 1:**
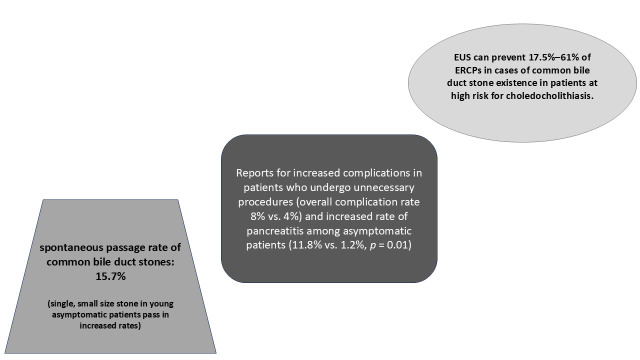
Graphical summary of spontaneous stone passage rates, ERCP complication rates, and potentially preventable ERCPs associated with an EUS-first strategy. ERCP = Endoscopic Retrograde Cholangiopancreatography; EUS = Endoscopic Ultrasound.

It is well established that both EUS and MRCP demonstrate excellent diagnostic performance for choledocholithiasis, with reported sensitivities and specificities of approximately 95% and 97% for EUS, and 93% and 96% for MRCP, respectively [5]. Accordingly, these modalities are widely utilized to guide the decision-making process regarding ERCP in patients with an intermediate probability of choledocholithiasis, as previously noted. However, EUS may outperform MRCP in detecting small stones, and it can be readily combined with ERCP during the same session to enable prompt diagnosis and therapy [8]. Recently, the EUS-first strategy, which aims to reduce unnecessary procedures in patients at high risk for choledocholithiasis, has gained attention. In a recent retrospective study, De Jong et al. [35] reported that, among 65 high-risk patients according to ESGE criteria, EUS prevented unwarranted ERCP in 11 of them (17.5%). Two additional studies investigated the role of an EUS-first strategy in patients with a high probability of choledocholithiasis and no evidence of CBDS on initial imaging [46,47]. As previously described, these patients carry a substantial risk for unnecessary procedures. Lin et al. [46] reported on a prospective study of 104 high-risk patients who had negative CT scan, with 44 following the EUS-first strategy and 60 undergoing ERCP directly. Overall, 61% of ERCPs were prevented in the first group. Moreover, the EUS-first strategy was more favorable in terms of both safety and cost-effectiveness. Similarly, a study by Patel et al. [47], involving 78 patients with high suspicion for choledocholithiasis and negative ultrasound, demonstrated that performing EUS prior to ERCP could avoid unnecessary ERCP in 57.7% of cases. A graphical summary illustrating spontaneous stone passage rates, ERCP complication rates, and potentially preventable ERCPs with an EUS-first approach is provided in Figure 1 to offer a practical and comprehensive overview.

### 2.10. Safety of Omitting ERCP

While the avoidance of unnecessary ERCPs is an important goal, it is equally vital to recognize the potential complications that may arise during the wait-and-see interval, particularly the development of acute cholangitis or acute pancreatitis from a retained bile duct stone. Andreozzi et al. [12] reported no unfavorable outcomes in the interval between the imaging diagnosis of choledocholithiasis and ERCP, even in patients who underwent the procedure more than 30 days after the diagnosis. Moreover, in another retrospective study, a wait-and-see approach for small stones (<5 mm) was found to be equally safe and more cost-effective compared with performing routine ERCP [48]. In contrast, a large retrospective cohort study demonstrated that among 3828 patients with choledocholithiasis, the rate of complications was significantly higher in those managed conservatively compared with those who underwent stone removal (25.3% vs. 12.7%, respectively) [49]. Of note, even small stones (<4 mm) were associated with a substantial complication rate of 15.9% when left in situ, whereas this rate was significantly lower (8.9%) among those who underwent removal [49].

In terms of the long-term safety of an EUS-first approach in patients with suspected choledocholithiasis, studies following patients for up to one year have shown comparable recurrence rates of biliary events. In a study including high risk for choledocholithiasis patients with a negative CT in which an EUS-first approach replaced immediate ERCP, rates of biliary events after three months—13.6% versus 15.0%—and cost advantages did not differ with the EUS-first strategy [46]. In another study including patients in the intermediate risk, authors have shown that after a period of approximately one year, no patients with a previous negative EUS had suffered a CBD-stone-related complication [50]. Similarly, Maruta et al. reported that, among 104 patients undergoing EUS as the initial assessment, only 1/42 patient (2.3%) with a negative EUS developed recurrent CBD stones within approximately one year, with no significant difference compared to 2/62 patients who underwent ERCP after a positive EUS (3.2%) [37]. These findings suggest that omitting ERCP in selected patients with suspected choledocholithiasis is safe and does not lead to increased long-term recurrence or complications.

When evaluating the safety of the EUS-based approach, the potential complications inherent to the procedure should also be considered. While bleeding, infection, and acute pancreatitis are significant adverse events associated with therapeutic EUS—particularly in the setting of EUS-guided fine-needle aspiration of pancreatic lesions—diagnostic EUS also carries a risk of complications. Mucosal injury of the upper gastrointestinal tract during echoendoscope insertion constitutes a clinically relevant risk, particularly in patients with coagulopathy. Moreover, although perforation is considered the most feared complication, it is extremely rare (incidence 0.03–0.06%) and typically occurs in anatomically narrowed segments such as the esophagus and the duodenal bend [51]. Finally, EUS is still associated with sedation and its own risks.

### 2.11. Critical Appraisal of the Evidence

Current evidence suggests that spontaneous stone passage is not an uncommon event, potentially resulting in unnecessary ERCPs and their associated risks. However, it is pivotal to address the gaps in the existing literature before drawing more reliable conclusions about the frequency of this event and the factors influencing it. First, most studies on this topic are retrospective, and none are randomized. Therefore, their results are susceptible to several significant types of bias and should be interpreted with caution. Moreover, all studies defined spontaneous stone passage based on findings at ERCP. As a result, only patients who ultimately underwent ERCP were included in incidence estimates, whereas those managed conservatively or reassessed using imaging modalities were excluded, thereby introducing potential selection bias. This approach may underestimate the true incidence of spontaneous stone passage, as cases resolving without endoscopic confirmation may remain unaccounted for. Conversely, a potential source of misclassification bias should also be considered. In some cases, the initial diagnosis of choledocholithiasis may be based on false-positive imaging findings. Therefore, a subsequent negative ERCP may not reflect spontaneous stone passage but rather the absence of a stone from the beginning, potentially leading to overestimation of its true incidence. In addition, studies assessing incidence show considerable heterogeneity, both in terms of study populations (e.g., clinical presentation, stone characteristics, etc.) and in the timing from diagnosis to intervention, which may contribute to the variation in reported incidence rates. To our knowledge, only one study focused on a relatively more specific patient group. Sanguanlosit et al. [20] found that among 96 patients with acute cholangitis, spontaneous stone passage occurred in 19.8%. Small stone size appears to be the most consistently reported factor associated with spontaneous stone passage, although proposed cut-off points differ between studies. A possible explanation for this variation could be the different imaging methods used to assess stone size across them. Another area of uncertainty concerns whether it is safe to omit ERCP in a patient with a known stone while awaiting the stone to migrate. While two studies reported that this approach was as safe as performing ERCP regarding the occurrence of biliary events during follow-up, Möller et al. [49], in their large retrospective study, demonstrated that the risk of complications was significantly higher when stones—even those <4 mm—were left in situ compared with when they were removed. Our review underscores the increased likelihood of unnecessary procedures in patients at high risk of choledocholithiasis and negative initial imaging. Nevertheless, one should bear in mind that classification into high-risk varies among studies based on either ASGE or ESGE criteria. These criteria differ from each other, with the former possibly being more specific based on limited data. Such variability in definitions may obscure the real incidence of unnecessary procedures in this setting.

## 3. Clinical Implications and Future Directions

Considering current evidence, a watchful waiting strategy may be discussed in specific scenarios where spontaneous passage of CBD stone is probable, and the risk of ERCP-related complications is non-negligible. For example, younger patients without a history of cholangitis, possessing a single small stone (<4 mm), and with a normal CBD diameter—especially if imaging was performed more than a week prior—may benefit from deferred ERCP with close follow-up. Moreover, an expectant approach may also be considered in patients with acute pancreatitis. Conversely, in older patients with multiple stones larger than 8 mm, CBD dilation, or cholangitis at presentation, the likelihood of spontaneous passage is low. Moreover, even if cholangitis is mild in severity, the risk of deterioration may surpass the risk of ERCP becoming complicated in these patients; thus, even a minimal delay in ERCP performance could be detrimental.

Before adoption of a conservative strategy is proposed, areas of uncertainty must be addressed. First of all, it should be further clarified whether leaving a CBD stone in situ can be considered safe in terms of recurrent biliary events, as the available data on this topic are contradictory. Moreover, an important remaining question besides in whom to wait, is how and for how long. The optimal follow-up duration, in which the likelihood of spontaneous passage is maximized without exposing patients to adverse outcomes from a retained stone, remains uncertain. Based on available data, a period of around three months, or approximately 90 days, appears reasonable, as emerging evidence suggests a passage rate of up to 90% within this timeframe [12]. Concerning how, it should be investigated whether continuous improvement in laboratory data—especially bilirubin levels—indicates spontaneous passage, or if imaging confirmation is mandatory. In the latter scenario, EUS or MRCP are considered the preferred options, with EUS demonstrating slightly higher accuracy than MRCP in detecting small CBD stones.

An EUS-first approach also seems reasonable in patients at high risk of choledocholithiasis who show no CBD stone on initial imaging. Although current guidelines support direct ERCP in this setting, our review also highlights a meaningful rate of unnecessary procedures within this group. In fact, an EUS-first strategy can avoid up to 61% of unnecessary procedures in high-risk patients and appears to be both safe and cost-effective.

Nevertheless, certain limitations of this approach warrant careful consideration. An important one is that not all medical centers have the capability to perform both EUS and ERCP. Moreover, despite the generally high performance of EUS in assessing the common bile duct, its accuracy is reduced for stones located near the hepatic hilum [52], potentially leading to misdiagnosis. Finally, beyond the aforementioned safety issues, it is noteworthy that EUS remains technically demanding for many endoscopists and, in order to achieve high diagnostic accuracy, should be performed by experienced operators. Preprocedural assessment of patient-related factors, such as the use of anticoagulants, along with careful management of sedation, is also essential for a successful outcome. Ultimately, due to the heterogeneity of existing studies and their methodologies, a universally validated, definitive algorithm for patient selection who should and can skip early ERCP, as well as precise recommendations regarding timing and the optimal imaging and laboratory methods for follow-up, remains elusive.

**Figure 2:**
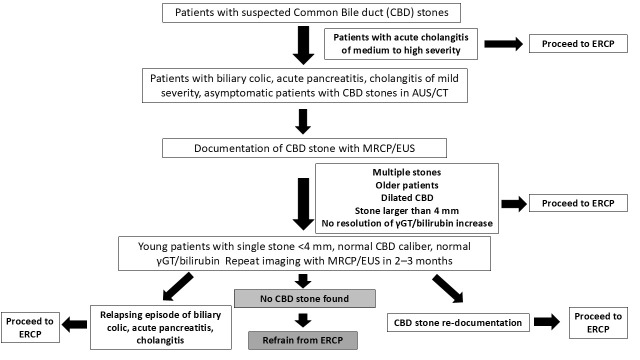
ERCP in patients with suspected common bile duct stones. ERCP = Endoscopic Retrograde Cholangiopancreatography; AUS = Abdominal Ultrasound; CT = Computed Tomography; MRCP = Magnetic Resonance Cholangiopancreatography; EUS = Endoscopic Ultrasound; γGT = gamma-glutamyl transferase.

Nonetheless, a provisional decision-making framework is illustrated in Figure 2, serving as a practical guide until further evidence becomes available.

## 4. Conclusions

Current evidence indicates that spontaneous stone passage is more likely in asymptomatic patients with small, single stones and a prolonged interval between diagnosis and intervention or in patients with acute pancreatitis. Therefore, a wait-and-see approach may represent an option in these scenarios. When deciding to omit ERCP, EUS may serve as a valuable tool to confirm whether the stone has migrated and to guide subsequent clinical decisions. It can also help guide management when a CBD stone is not visualized on initial imaging but there remains a high suspicion of choledocholithiasis based on clinical and laboratory data. In this setting, the EUS-first protocol can lead to a significant reduction in unnecessary ERCPs, while improving cost-effectiveness. However, additional data on the safety of a conservative approach—especially in the context of a known CBD stone—are required to better define its role. Moreover, technical aspects such as the optimal timing for assessing stone migration should be further clarified. Expanding the availability of EUS is important to enable wider implementation of an EUS-based strategy. Last but not least, the decision whether to proceed with ERCP or to omit it should be made in a patient-specific manner, in which patient-related factors (comorbidities and age) and operator-specific factors (expertise, availability, and anesthesiological support) must also be taken into account.

## Data Availability

No new data were created or analyzed in this study.
